# Connexins-Based Hemichannels/Channels and Their Relationship with Inflammation, Seizures and Epilepsy

**DOI:** 10.3390/ijms20235976

**Published:** 2019-11-27

**Authors:** Laura Medina-Ceja, Juan C. Salazar-Sánchez, Jorge Ortega-Ibarra, Alberto Morales-Villagrán

**Affiliations:** 1 Laboratory of Neurophysiology, Department of Cellular and Molecular Biology, CUCBA, University of Guadalajara, Guadalajara, Jalisco 45110, México; ss_juan_carlos@hotmail.com; 2 MexBio Research Innovations S.A. de C.V., El Salto, Jalisco 45696 México; jaguarjmo@gmail.com (J.O.-I.); amorales56@gmail.com (A.M.-V.)

**Keywords:** connexins, epilepsy models, gliotransmitters, interleukins, neuroinflammation, seizures

## Abstract

Connexins (Cxs) are a family of 21 protein isoforms, eleven of which are expressed in the central nervous system, and they are found in neurons and glia. Cxs form hemichannels (connexons) and channels (gap junctions/electric synapses) that permit functional and metabolic coupling between neurons and astrocytes. Altered Cx expression and function is involved in inflammation and neurological diseases. Cxs-based hemichannels and channels have a relevance to seizures and epilepsy in two ways: First, this pathological condition increases the opening probability of hemichannels in glial cells to enable gliotransmitter release, sustaining the inflammatory process and exacerbating seizure generation and epileptogenesis, and second, the opening of channels favors excitability and synchronization through coupled neurons. These biological events highlight the global pathological mechanism of epilepsy, and the therapeutic potential of Cxs-based hemichannels and channels. Therefore, this review describes the role of Cxs in neuroinflammation and epilepsy and examines how the blocking of channels and hemichannels may be therapeutic targets of anti-convulsive and anti-epileptic treatments.

The goal of the present review is to clarify the importance of connexins (Cxs) in Cxs-based channels (gap junctions/electric synapses) and hemichannels (connexons), in neuroinflammation, and their relationship with seizures and epilepsy. This review is focused on classic and up-to-date studies of seizure and epilepsy models, to which neuroinflammation and Cxs-based channels and hemichannels are relevant. The first part of this review takes into account information about the molecular and cellular characteristics of Cxs-based channels/hemichannels, as well as their principal functions in neurons and glial cells. The second part describes their relationship with neuroinflammation. The third part discusses how some blockers of channels and hemichannels may be therapeutic targets of anti-convulsive and anti-epileptic treatments, in different models of seizures and epilepsy.

## 1. Molecular and Cellular Characteristics of Connexins-Based Hemichannels and Channels

Connexins (Cxs) are a family of 21 protein isoforms, and some studies have shown that some of these structural proteins in Cx-based channels and hemichannels have an average half-life of 2–3 h [1,2]. Of these isoforms, eleven of them are expressed in the central nervous system (CNS), and are found in neurons and glia. The principal Cxs associated with neurons are Cx45 and Cx36 [3]. In astrocytes, Cx43 is the most important Cx, but Cx26 and Cx30 are also expressed [3,4,5]; Cx29, Cx32, Cx45, and Cx47 are expressed in oligodendrocytes [6,7], and Cx32, Cx36, and Cx43 are expressed in microglia [8,9,10].

Homomeric connexons or hemichannels are formed by six of the same Cx isoforms, and with different Cx isoforms, form a heteromeric connexon or hemichannel; two hemichannels in two neighboring cells form a gap junction channel with an aqueous pore diameter of 1 nm [11], and charged surface walls that depend on the Cx type [12]. These channels, through which cells are electrically and chemically coupled, can be formed from homomeric or heteromeric hemichannels, and are therefore called homotypic or heterotypic channels, respectively. The conductance of these Cxs-based channels and hemichannels is regulated by calcium concentration [13,14], intracellular pH [15,16,17,18], some neurotransmitters, such as serotonin and dopamine [19,20], the trans-junctional voltage (relative voltage difference between coupled cells), and the membrane voltage [21,22]. The conductance is related to the type of Cxs that make up the channels and hemichannels; for example, Cx36 has a conductance of 10–15 pS, while Cx45 and Cx43 have a conductance of 27.84 ± 0.25 pS [23,24,25] and 36.8 ± 0.54 pS [25], respectively.

Experimental evidence has demonstrated the presence of Cxs-based channels and hemichannels in important brain regions susceptible to seizure generation, such as the hippocampus, amygdala, and neocortex [3,26,27,28,29,30]. Cxs-based channels are localized between the axons of principal neurons [31], between interneurons with a principally dendrodendritic localization [26], and in neurons with mixed chemical/electrical synapses [32,33]. The principal function of Cxs-based hemichannels is the release of gliotransmitters via a Ca^2+^-dependent mechanism [13], while channels participate in the propagation of Ca^2+^ waves, with long-range coupling of the astrocytic network [5]; in neurons, they participate in the synchronization and generation of oscillations in the gamma range (through interneurons dendrites) and ripple frequency (through pyramidal cell axons) [30,31].

## 2. Connexins-Based Hemichannels/Channels and Neuroinflammation

**Figure 1:**
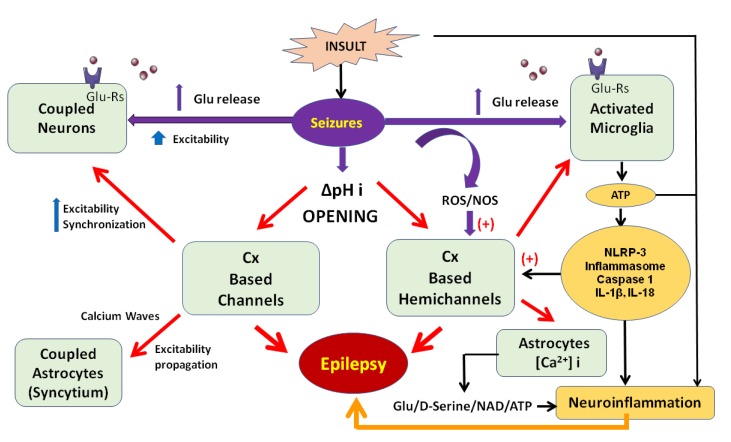
General representation of the connexins (Cxs)-based channels and hemichannels participation in seizures and epilepsy, through synergistic actions of neuroinflammation and hyper excitability and synchronization. Different types of insults can produce seizures that modify intracellular pH, which opens hemichannels in glial cells (activated microglia and astrocytes), with the consequent calcium-dependent release of gliotransmitters (Glutamate Glu, D-serine, NAD and ATP) and proinflammatory molecules (NLRP-3 inflammasome: Caspase 1, IL-1β, IL18) that lead to neuroinflammation and open Cxs-based hemichannels. This last action is also facilitated by reactive oxygen species (ROS) and reactive nitrogen species (NOS) produced during seizures. In the same way, astrocytes can reinforce seizures through the opening of Cxs-based channels that activate a calcium wave in an astroglial syncytium that propagates excitability. In neurons, the opening of channels increases abnormal high excitability and synchronization that reinforces the cycle of seizures generation.

Neuroinflammation is the response to injury, the normal aging process, dementia, stroke, hypertension, depression, diabetes, tumors, infections, toxins, drugs, acute trauma, or neurodegenerative disease that involves the coordination of inflammatory cells, and biochemical activities that occur in the CNS [34,35,36,37]. Events associated with neuroinflammation include the activation of astrocytes and microglia, the participation of oligodendrocytes and other nervous system cells, the release of cytokines and chemokines, increased levels of prostaglandins, the infiltration of cells from the bloodstream, and the generation of reactive oxygen (ROS) and nitrogen species (NOS) (Figure 1) [38,39,40]. 

Neuroinflammation is regulated by components of the immune system, and by pathogen recognition receptors (PRRs) [41]. Similarly, the entry of calcium into the cell through the activity of channels and hemichannels positively regulates neuroinflammation [42], which is a common consequence of epileptic seizures, and the pathogenesis of some types of acquired and genetic epilepsy [43].

The binding of pathogen-associated molecular patterns (PAMPs) or damage-associated molecular patterns (DAMPs) to PRRs causes the activation of signaling pathways that result in neuroinflammation [44]. Some DAMPS include ATP, interleukin (IL)-1β, IL6, IL11, IL15, IL17, TNFα, uric acid, and high-mobility protein group 1 (HMGB1) [45,46]. In this sense, Cxs plays an important role in the generation and maintenance of neuroinflammation, because hemichannels have little selectivity, and their opening is considered harmful since neurotoxic damage can be caused by the induced release of gliotransmitters, ATP, glutamate, and other molecules (Figure 1) [47].

Hemichannels formed by Cxs are activated by the presence of proinflammatory cytokines (TNFα and IL1β) and ROS [48] through increases in the concentrations of intracellular Ca^2+^ and extracellular K^+^ and ATP, as well as decreases in the extracellular concentration of Ca^2+^ and the redox potential [49,50]. Then, under stressful conditions, Cxs-based hemichannels amplify the damage and induce neuroinflammation [51]. However, once inflammation is established, positive feedback that promotes increased opening of Cxs-based hemichannels is produced [52]. The opening of Cxs-based channels and hemichannels due to inflammation begins a positive feedback cycle that, among other things, results in excessive ATP release [53].

It is recognized that inflammation is activated by two signals. Of these signals, one is mediated by the activation of PRRs through PAMPs or DAMPs that result in the induction of the NF-κB pathway, which promotes the expression of pro-IL-1β, and genes associated with inflammatory proteins [54,55]. The second signal is attributed to ATP [56,57,58] and glutamate via N-methyl-D-aspartate (NMDA) receptors [59,60]. For example, it has been observed that, in astrocytes, the activation of exogenous ATP-induced inflammation triggers an increase in IL-1β production [61]. 

Under neurodegenerative conditions, neuroinflammation involves the primordial participation of CNS cells, including microglia, oligodendrocytes, and astrocytes [62,63]. Astrocytes were first described by Dieters in 1865, and named by von Lenhossék in 1895, they are currently known as the most abundant cell type in the CNS [64,65]. Astrocytes participate in a large number of functions, including those related to pro- and anti-inflammatory processes, that either contribute to inflammation [66,67,68] or respond to it [69], and in epilepsy [70]. The expression of Cx43, and in lower quantities Cx26 and Cx30, of channels and hemichannels in astrocytes is relevant to neuroinflammatory processes because it allows both the exchange of small molecules [71], and gliotransmission [72,73,74]. 

Neuroinflammation induces the activation of two types of reactive astrocytes, called A1 and A2, as determined by transcriptome analysis. It has been suggested that the activation of A1 by NF-κB [75,76], but not the activation of A2 has reinforcing effects on inflammation. Additionally, reactive astrocytes increase the expression of genes responsible for the formation of synapses that can, nevertheless, lead to epilepsy [77]. Another relevant feature of astrocytes is their high degree of interconnection through Cxs-based channels, which create large functional syncytial networks, that are electrically and metabolically coupled, through which network Ca^2+^ waves can propagate excitability [78,79]. 

Oligodendrocytes are small myelin-forming cells that are widely distributed in the CNS, and based on morphology, can be divided into four subtypes [80]. They mainly contribute to conduction inside neuronal circuits, but they also participate in metabolic supply and ion buffering [81,82]. Oligodendrocytes mainly express Cx29, Cx32, and Cx47; through these connections, they form channels with astrocytes and with themselves (heterotypic unions of Cx30 and Cx32, and of Cx43 and Cx47) [7,83]. These functional unions of Cxs even participate in the release of gliotransmitters, although it is not clear if oligodendrocytes have the necessary molecular machinery to fulfill this task [84]. It has also been suggested that oligodendrocytes may be associated with acquired neuroinflammation [78], but more robust information, as well as increased information on the involvement of oligodendrocytes in neuroinflammation, is needed.

Microglia are tissue-resident innate immune cells of the CNS that constitute approximately 5–10% of all CNS cells [85]. The resting state or non-activated state of microglia [86] may result from signals, such as transforming growth factor-β (TGFβ), an anti-inflammatory cytokine that is constitutively expressed in the CNS in the non-inflamed brain [87]. Microglia, particularly those that focus on debris clearing and the elimination of dead cells and synapses are essential in the maturation of neuronal circuits [88], the development of synaptic plasticity [89], and CNS homeostasis [90,91]. In the resting state, microglia exhibit their own Cxs profile, which involves the expression of Cx32 [92] and Cx36 [93,94]; however, Cx43 is not expressed by microglia [95,96,97]. 

Under neurological conditions such as epilepsy, microglia are rapidly activated, release proinflammatory cytokines, take on an amoeboid morphology, express a large number of surface receptors, and can detect DAMPs, which are recognized by PRRs [98,99,100,101], all of these can lead to neuronal hyperexcitability and neurodegeneration. Several studies have shown an increase in the expression of Cx32 [102], Cx36 [103], and Cx43 in microglia, which has been correlated with the development of chronic degenerative diseases. Additionally, it has been shown that, during the establishment of neurological damage, such as seizures and epilepsy, ATP is released mainly by the Cxs of astrocytes, and the pannexins of oligodendrocytes [104,105]. In vivo and in vitro studies have shown that the excessive release of ATP and glutamate is toxic to the CNS [104,105,106]; subsequently, ATP is recognized by P2x7 microglial receptors, and their activation causes potassium outflow, which is a critical event for the activation of the NLRP3 inflammasome. A central framework receptor, called nucleotide-binding oligomerization domain-like receptor family pyrin domain containing 3 (NLRP3) that initiates signaling, and three key protein organization-based proteins, results in the recruitment of procaspases and caspases (Figure 1) [107]. The assembly of the NLRP3 inflammasome results in the conversion of procaspase-1 from its pro form to its active form, which then processes the cleavage of several substrates, such as pro-IL-1β and pro-IL-18, in the mature cytokines IL-1β and IL-18 [108]; this process can aggravate inflammation. In addition, numerous studies have implicated neuroinflammation as the cause, and result, of epilepsy [109,110].

## 3. Blockers of Connexins-Based Channels/Hemichannels as Anticonvulsive and Antiepileptic Therapeutic Targets in Different Models of Seizures and Epilepsy

Metabolic, mechanical, or genetic insults, as well as an imbalance between γ-Aminobutyric Acid (GABA) and glutamate, can generate seizures through the excessive release of glutamate [111], and the overactivation of their receptors in neurons and astrocytes; this results in an increase in intercellular calcium concentrations in both types of cells [112,113,114]. However, the effect of this activation and association with Cxs-based channels and hemichannels can establish prolonged seizures and epilepsy through different paths in neurons and glial cells (Figure 1).

In neurons, it can drive hyperexcitability and synchronicity as a consequence of intense neuronal firing between pyramidal cells of the hippocampus that is sustained by the uncontrolled opening of axo-axonal Cxs-based channels, induced by pH changes (alkalinization) [31,115,116,117], or be as observed in dendritic Cxs-based channels with strong olivary coupling, after the activation of NMDA receptors in inferior olive neurons [118,119]. Additionally, these observations support the chemical modulation of Cxs-based channels and hemichannels by neurotransmitters, such as glutamate or even serotonin (5-HT) [19]. The pathological alkaline-acidosis environment generated during and after seizures facilitate the conductance of Cxs-based channels that strengthens neuronal coupling in networks, which induces increased synchronization and excitability (Figure 1) [120,121,122,123,124].

These facts can be assessed by in vitro and in vivo pharmacological experiments, in which the blockade of Cxs-based channels or hemichannels, has been shown to have anti-convulsive effects (Table 1). These studies used non-specific blockers such as carbenoxolone, quinine, and mefloquine, and the combination of these drugs with classic anti-convulsants [125,126,127,128,129,130,131,132,133]; carbenoxolone blocks Cxs indistinctly via the phosphorylation or internalization of Cxs subunits [134,135,136,137], quinine blocks not only Cx36- and Cx50-based channels, but also ion channels associated with the electric membrane properties of neurons [134,135,136,137,138,139,140,141], and mefloquine has effects on different neurotransmitter systems, acting as a GABA A antagonist or inhibiting 5-HT3 receptors [142]; it induces short action potentials and has effects on l-type calcium channels [143], in some cases increasing spontaneous excitatory currents [144].

However, studies in which more effective drugs, including mimetic peptides such as Gap 26 (sequence: VCYDKSFPISHVR) and Gap 27 (sequence: SRPTEKTIFI), were used to block Cxs-based channels or hemichannels showed similar results [145,146,147]; in this respect, Gap 27, a specific peptide to the extracellular loop domain of Cx43, Cx40, and Cx37, decreased recurrent epileptiform activity in hippocampal slices [146], and prevented neuronal death caused by seizures induced by the GABA-A receptor antagonist bicuculline [147].

The pathological cycle of hyperexcitability and synchronization can trigger epileptogenesis, and Cxs-based channels and hemichannels play an important role in this process, since some studies have shown their participation in fast ripples, a high frequency oscillation that is considered an electrophysiological marker of epileptogenicity [148,149]. Several studies have demonstrated that fast ripples are decreased by blockers of Cxs-based channels and hemichannels in the hippocampus of animals with pilocarpine-induced epilepsy [150,151], as well as in vitro and in silico [152]. These facts indicate their importance and how hypersynchronization and excitability of neurons synergistically with neuroinflammation maintain pathological conditions in networks that address cerebral damage and cell loss, which favor epileptogenesis.

In glial cells (astrocytes and microglia), after the activation of glutamate receptors (NMDA, AMPA and metabotropic glutamate receptors), gliotransmitters such as ATP, glutamate, Nicotinamide Adenine Dinucleotide (NAD), and d-serine are released through Cxs-based hemichannels, which sustain an excitatory extracellular environment through the activation of extrasynaptic NMDA receptors in neurons of the hippocampus (CA1), and together with astrocytic calcium waves in networks, propagate neuronal activity, increasing the excitability and synchronicity of surrounding neurons (Figure 1), as observed in various pharmacologic in vitro studies [146,153,154,155,156]. Additionally, as described previously, the release of gliotransmitters can modulate the inflammatory response, activate immunological cells, and synergistically trigger irreversible neuronal damage [157]. Moreover, Cxs-based hemichannels can support epileptic activity by supplying glucose to epileptic neuronal networks [158].

There are specific pharmacological blockers of Cxs-based hemichannels, such as peptide 5 (with a short incubation time or at a low concentration), Gap 19, and La^3+^, that have been used in a few studies to support the principal role of hemichannels in seizures, and to demonstrate their anti-convulsive effects in in vitro models [159,160], and in some animal models of seizures [160]. In this last study, the suppression of seizures was associated with a decrease in d-serine concentration. Despite the importance of Cxs-based hemichannels in seizures and epilepsy (Table 1), there have been few pharmacological studies that have demonstrated the use of these drugs as a new therapeutic strategy against seizures and epilepsy. Accordingly, it is necessary to continue researching this topic experimentally, to further understand their potential as anti-convulsive and anti-epileptic treatments.

**Table 1:** Anticonvulsive effects of principal blockers of Cxs-based hemichannels and channels in different in vitro and in vivo models of seizures and epilepsy.

Blocker(s) of Cxs-Based Hemichannels or Channels	Seizure or Epilepsy Model	Technique/Brain Region	Main Results	Citation
Carbenoxolone (10 μM) and quinine (35 μM) administered through a piece of filter paper covering the cortical surface	In vivo: local application of crystalline 4-AP on the surface of the cortex	Electrocorticography (ECoG) in the brains of adult Wistar rats (male and female, 30–40 days old, 200–250 g)	Anticonvulsive effect of carbenoxolone (reduced the generation of seizure discharges); quinine decreased summated ictal activity and the amplitudes of seizure discharges	[125]
Carbenoxolone (150 mM) and meclofenamic acid (50 mM) administered through a cannula implanted in the right motor cortex	In vivo: a model of refractory focal cortical epilepsy induced with tetanus toxin (50 ng/0.5 μL, pH 7.5) in 2% bovine serum albumin	Intracranial electroencephalography (iEEG) in the right motor cortex of adult Sprague-Dawley rats (240–320 g)	Reduced the percentage of seizure time	[126]
Quinine (200, 400 or 1000 nmol) administered through a cannula implanted in the ventricle of the brain	In vivo: a model of epilepsy induced by 300 IU of crystallized penicillin	Epidural EEG in adult Wistar rats (male, 4 months)	Decreased the amplitude and frequency of epileptiform spikes and attenuated convulsive behavior	[127]
Carbenoxolone injection (50 nmol) administered through a cannula implanted in the entorhinal cortex	In vivo: a model of seizures induced by 4-aminopyridine (10 nmol) administered through a cannula implanted in the entorhinal cortex	Epidural EEG and iEEG in the entorhinal cortex of adult Wistar rats (male, 250–350 g)	Decreased the amplitude and frequency of epileptiform discharges and the number and duration of epileptiform trains	[128]
Carbenoxolone, Gap 27 (mimetic amino acid residues 201–211, SRPTEKTIFII) and SLS (amino acid residues 180–195, SLSAVYTCKRDPCPHQ) peptides	In vitro: epileptiform activity induced in organotypic hippocampal slice cultures by stimulation	Extracellular recordings from the CA1 and CA3 regions of hippocampal slices from 7-day-old Wistar rats	Carbenoxolone inhibited both spontaneous and evoked seizure-like events; the Cx43 mimetic peptides selectively attenuated spontaneous recurrent epileptiform activity after prolonged (10 h) treatment	[146]
Quinine injection (35 pmol) administered through a cannula implanted in the entorhinal cortex	In vivo: a model of seizures induced by 4-aminopyridine (10 nmol) administered through a cannula implanted in the entorhinal cortex	Epidural EEG and iEEG in the entorhinal cortex of adult Wistar rats (male, 250–350 g)	Decreased the amplitude and frequency of discharge trains and blocked seizure behavior in five of six rats	[129]
Cx43 mimetic peptide (5 and 50 μM, sufficient to block hemichannels, VDCFLSRPTEKT, extracellular loop two of Cx43)	In vitro: a model of epileptiform injury induced by bicuculline methochloride (BMC) (48 h exposure to 100 μM) in hippocampal slices cultures from 6- to 8-day-old Wistar rats	Measurement of cell death after epileptiform activity (fluorescence signal) and immunohistochemistry for microtubule-associated protein (MAP2)	Exerted a protective effect in the CA1 region during the recovery period (24 h after BMC treatment)	[147]
Carbenoxolone (20 mg/kg, i.p.) once a day for 14 days	In vivo: a model of posttraumatic epilepsy induced by ferric ions (microinjection of 10 μL of 0.1 M FeCl_3_ solution into the sensorimotor area)	Evaluation of convulsive behavior according to the Racine scale in adult male Sprague-Dawley rats aged 6–8 weeks and weighing 220–250 g	Ameliorated convulsive behavior score in rats	[130]
Carbenoxolone (50 nmol) and quinine (35 pmol) administered through a guide cannula in the entorhinal cortex (0.2 μL/min for 5 min)	In vivo: a pilocarpine-induced model of temporal lobe epilepsy (1.2 mg/μL pilocarpine hydrochloride in a total volume of 2 μL, intracerebroventricular (i.c.v.)	iEEG in the hippocampus of epileptic adult Wistar rats (male, 190–200 g)	Decreased the number of Fast Ripples (FR) events and oscillation cycles per FR event	[150]
Carbenoxolone (0.2 mM)	In vitro: Neocortical slices	Neocortical slices from epileptic patients (temporal and occipital regions)	Strongly decreased the incidence of FR events	[152]
	In silico: a small network of 256 multicompartment cells	Simulated networks containing only pyramidal cells, coupled only by axonal gap junctions, and without chemical synapses or interneurons	The network produced FR events via a cluster of axonal Cx-based channels (gap junctions)	
Carbenoxolone (40 mg/kg, i.p.) and carbenoxolone + valproic acid (300 mg/kg, i.p.)	In vivo: a kindling model of epilepsy induced by pentylenetetrazole (35 mg/kg, i.p.)	Epidural EEG in Wistar rats (female, 12–15 weeks old, 200 ± 50 g)	Carbenoxolone prevented generalized seizures and reduced seizure stage, seizure duration and spike frequency; no significant difference between carbenoxolone + valproic acid and valproic acid	[131]
Carbenoxolone (50 mg/kg, i.p., for 3 days) and quinine (50 mg/kg, i.p. for 3 days)	In vivo: a lithium/pilocarpine-induced *Status epilepticus* (SE) model (i.p. injection of 50 mg/kg pilocarpine 18–20 h after the i.p. injection of 127 mg/kg lithium chloride)	iEEG in the hippocampus of adult Sprague-Dawley rats (male)	Reduced the spectral power of FR events 10 min after SE	[151]
Coadministration of valproate (VPA), phenytoin (PHT), or carbamazepine (CBZ) at subtherapeutic doses (i.p.) with carbenoxolone (60 mg/kg, i.p., 5 mL/kg) or quinine (40 mg/kg, i.p., 5 mL/kg)	In vivo: maximal electroshock (MES)-induced (frequency of 60 Hz, pulse width of 0.6 ms, shock duration of 0.6 s, and a current of 90 mA) and pentylenetetrazole (PTZ)-induced (70 mg/kg, i.p.) models of seizures	EEG and power spectral analysis in Wistar rats (male, 270–300 g)	Quinine increased the anticonvulsant activity of VPA, PHT and CBZ to generalized tonic-clonic seizures in the MES-induced model and the anticonvulsant activity of CBZ only to generalized tonic-clonic seizures in the PTZ-induced model	[132]
TAT-Gap 19 (200 mM for in vitro experiments; 12 mM intrahippocampal and 1 mM in a total volume of 1 μL i.c.v. for in vivo experiments); TAT-Gap19 (25 or 50 mg/kg i.p., electrical stimulation for in vivo experiments)	In vitro: pilocarpine (15 μM) administration in acute brain slices; In vivo: pilocarpine model in mice and rats (12 mM, intra hippocampal) Limbic psychomotor seizures by corneal stimulation	In vitro: ethidium bromide uptake experiments in acute brain slices from Glial Fibrillary Acidic Protein-enhanced Green Fluorescent Protein (GFAP-eGFP) transgenic mice (both genders, 2 months old); In vivo: video-EcoG analysis (seizure duration) or modified Racine’s scale evaluation (to score kindling-induced behavioral changes) in NMRI mice (male, 20–30 g) and, video-EcoG and Racine scale evaluation of convulsive behavior in Wistar rats (male, 250–300 g)	In vitro: dye uptake experiments demonstrated that astroglial Cx43 hemichannels open in response to pilocarpine, and this was inhibited by TAT-Gap19. In vivo: TAT-Gap19 suppressed seizures and decreased D-serine concentrations; these effects were reversed by exogenous D-serine administration, and a similar effect was observed for the electrical stimulation model	[160]
In vitro experiments: Carbenoxolone (200 mM) quinine (100 mM) and La(NO_3_)_3_ (a blocker of Cx-based hemichannels) In vivo experiments: carbenoxolone (100 mg/kg) and quinine (40 mg/kg)	In vitro: low-Mg2+-induced epilepsy model In vivo: Wistar Albino Glaxo/ Rijswijk (WAG/Rij) rat model of absence epilepsy	Field potential recordings, evaluation of seizure-like events (SLEs) in hippocampal-entorhinal slices from Wistar rats (11–14 days old); epidural EEG (frontal and parietal cortex) in WAG/Rij rats (female, 11–12 months old, 195–210 g); recordings of bilaterally synchronous spike-wave discharges (SWDs)	Carbenoxolone prevented the occurrence of SLEs and aggravated seizures in non-convulsive absence epilepsy; quinine did not prevent SLEs but increased the number and total time of SWDs and decreased the length of the interictal intervals; La^3+^ completely abolished SLEs	[159]

## 4. Conclusions

There is evidence regarding the relationship between Cxs-based channels and hemichannels on seizures and epilepsy in different models, in which the uncontrolled opening of these channels by pH changes as a consequence of intense neuronal firing sustains inflammation, hyperexcitability, and synchronicity that maintain this pathological condition. The use of some blockers of Cxs-based channels and hemichannels may be therapeutic agents for the treatment of seizures and epilepsy.
